# Editorial: Roles of non-coding RNAs in infectious diseases - volume II

**DOI:** 10.3389/fcimb.2023.1202616

**Published:** 2023-04-25

**Authors:** Bikash R. Giri, Guofeng Cheng

**Affiliations:** ^1^ Shanghai Tenth People’s Hospital, Institute for Infectious Diseases and Vaccine Development, Tongji University School of Medicine, Shanghai, China; ^2^ Department of Zoology, Utkal University, Bhubaneswar, India

**Keywords:** infectious diseases, non-coding RNA, miRNA, lncRNA, Chagas disease, acute-on-chronic liver failure, *Flavivirus*, *Mycobacterium tuberculosis*

This editorial summarizes the contributions to the Frontiers Research Topic “*Roles of Non-coding RNAs in Infectious Diseases*” established under the Frontiers in Cellular and Infection Microbiology. Pathogenic organisms, namely bacteria, viruses, and parasites, cause infectious diseases that can spread directly or indirectly among individuals. This results in significant public health challenges and substantial economic losses to the veterinary industry. Recent studies suggested that non-coding RNAs, such as microRNAs (miRNAs), long noncoding RNA (lncRNAs), and circular RNAs (circRNAs), can regulate pathogen infection and pathophysiology. Deep understanding of non-coding RNA roles in pathogenicity and host defence may lead to developing novel intervention strategies against infectious diseases. The Research Topic provides the perspective of the pathogenicity, and biomarker potential of non-coding RNAs in various infectious diseases, including host pathological responses and pathogen-host interactions, through basic and applied research. This editorial contains 5 publications on this Research Topic, covering studies on the roles of small and long non-coding RNAs including miRNAs and lncRNAs in modulating host immune response and pathogen-host interactions.

The protozoa parasite *Trypanosoma cruzi* causes Chagas disease considered a tropical neglected parasitic disease resulting in high morbidity and mortality globally. The study titled “*Circulating MicroRNAs and myocardial involvement severity in chronic Chagas cardiomyopathy*” Gómez-Ochoa et al. aimed to identify the potential role of microRNAs in the severity of Chagas cardiomyopathy. Chronic Chagas cardiomyopathy (CCC) is a cardiac manifestation of the disease and is a leading cause of heart failure in Latin America. The study included 74 patients and assessed the association of six circulating microRNAs such as miR-34a-5p, miR-208a-5p, miR-185-5p, miR-223-5p, let-7d-5p, and miR-454-5p with echocardiographic variables. The study showed an association between higher miR-223-5p levels with better-left ventricle ejection and lower NT-proBNP levels. The present study demonstrated the potential link between low levels of miR-123-5p with CCM worsening with signalling pathways related to receptor tyrosine kinases. The identified microRNA may play an important role in the pathogenesis of CCC, and further studies are needed to investigate their potential as therapeutic targets for CCC.

Acute-on-chronic liver failure (ACLF) accounts for acute liver function deterioration and often occurs in patients infected with hepatitis B virus (HBV). The next publication, “*Plasma-derived exosomal SncRNA as a promising diagnostic biomarker for early detection of HBV-related acute-on-chronic liver failure*” was designed to identify the role of small non-coding RNAs in early diagnosis of ACLF related to hepatitis B virus (Xu et al.). The researchers isolated exosomes from the plasma of 4 chronic patients with flare, 6 HBV-ACLF patients and 3 normal subjects and submitted them for small noncoding RNA sequencing. The differentially expressed small non-coding RNAs were verified in a validated cohort of 313. The sncRNAs, hsa-miR-23b-3p, has-miR-223-3p, has-miR-339-5p, tsRNA-20, tsRNA-46, and rsRNA-249 were explicitly differentially expressed in plasma exosomes of HBV-ACLF. Based on these results the researchers developed a diagnostic model on the levels of these sncRNAs, which showed high sensitivity (94.80%) and specificity (100%) for diagnosing ACLF related to HBV. The study’s findings suggest that plasma-derived exosomal sncRNAs have the potential to serve as promising diagnostic biomarkers for the early detection of HBV-related ACLF. These findings may contribute to developing more effective and timely interventions for patients with HBV-related ACLF.

Genus *Flavivirus* is a family of positive, single-stranded, enveloped RNA viruses and includes several important viruses such as Dengue, Zika, West Nile, Japanese encephalitis, Murray Valley encephalitis, tick-borne encephalitis, Yellow fever, Saint Louis encephalitis, Usutu viruses. The review titled “*Interactions of host miRNAs in the flavivirus 3´UTR genome: From bioinformatics predictions to practical approaches*” Avila-Bonilla and Salas-Benito compiled the available information on host miRNAs and the 3’ untranslated region (UTR) of the flavivirus genome. The review described several host miRNAs, including miR-484 and miR-744, targeted the stem loop of the 3’ UTR of all four DENV serotypes and the over-expression DENV-2 NS1 protein production. Similarly, miR-548g-3p interacts with the stem loop A in the dengue virus genome’s 5’ UTR, down-regulating the viral RNA accumulation and protein expression in U937 cells. Furthermore, the researchers proposed that treatment with antagomiRs, synthetic inhibitors of miRNA function, could increase the expression of the viral genome *in vitro*. The review suggests that host miRNAs regulate flavivirus replication by interacting with the 3’ UTR of the viral genome that may represent the novel miRNA-based strategies for preventing flavivirus infections.

LncRNAs are associated with the regulation of host immunity during porcine infection disease. The study titled *“RNA-seq reveals a novel porcine lncRNA MPHOSPH9-OT1 induces CXCL8/IL-8 expression in ETEC infected IPEC-J2 cells”* from Jiang et al. aimed to identify the role of a novel porcine long non-coding RNA (lncRNA) called MPHOSPH9-OT1 in the immune response of intestinal epithelial cells to enterotoxigenic *E. coli* (ETEC) F49 infection. The researchers used RNA sequencing to analyse the gene expression profiles of porcine intestinal epithelial cells (IPEC-J2) infected with ETEC and identified 10150 novel porcine lncRNAs, leading to the identification of 161 differentially expressed lncRNAs (65 upregulated and 96 downregulated) associated with cell growth and inflammation-related pathways. They observed that the pro-inflammatory chemokine CXCL8/IL-8 expression positively correlated with the expression of MPHOSPH9-OT1. The study’s findings suggest that the novel porcine lncRNA MPHOSPH9-OT1 plays a role in the immune response of intestinal epithelial cells to ETEC infection by inducing CXCL8/IL-8 expression. This may contribute to developing more effective strategies for preventing and treating ETEC-induced enteric diseases in pigs.

MiRNA expression patterns are distinct in *Mycobacterium tuberculosis* (Mtb)-infected patients compared to healthy people. The study titled *“microRNAs associated with the pathogenesis and their role in regulating various signalling pathways during Mycobacterium tuberculosis infection”* by Chauhan and Davuluri aimed to investigate the role of miRNAs in the pathogenesis of Mtb infection and their involvement in regulating various signalling pathways. The researchers comprehensively reviewed the literature on miRNAs associated with Mtb infection and their role in regulating multiple signalling pathways. They highlighted that several miRNAs are dysregulated during Mtb infection (upregulated miRNAs: miR-125a, miR-155, miR-31, miR-99b, miR-194, miR-21-5p, miR-223-3p, miR-2909, miR-26b, miRNA-124, miRNA-23a-5p; miRNA-1178, miR-325-3p, miR-148a etc; downregulated miRNAs: miR-27b, miR-708-5p, miR-146a, miR-146a-5p, miR-18b-5p, miR-140 etc), and their expression is associated with the progression and severity of the disease. The review also summarized the role of miRNAs in regulating several signalling pathways involved in Mtb infection, immune escape, replication, apoptosis, autophagy and host lipid metabolism. The researchers also discussed the role of miRNAs as diagnostic markers for Mtb infection. These findings may contribute to developing new diagnostic and therapeutic strategies for Mtb infection.

## Author contributions

All authors listed have made a substantial, direct, and intellectual contribution to the work and approved it for publication.

